# Contamination Landscapes: Spatio-Temporal Record and Analysis of Pathogens in Clinical Settings

**DOI:** 10.3390/ijerph20031809

**Published:** 2023-01-18

**Authors:** Christoph Höser, Thomas Kistemann

**Affiliations:** 1 Institute for Hygiene and Public Health, University Hospital Bonn, D-53127 Bonn, Germany; 2 Department of Geography, University of Bonn, D-53115 Bonn, Germany; 3 Center for Development Research, University of Bonn, D-53113 Bonn, Germany

**Keywords:** hospital acquired infections (HAI), nosocomial outbreaks, infection chain, contamination landscape

## Abstract

Nosocomial outbreaks require quick epidemiological clarification of possible chains of infection, since the pathogen usually has a head start that has to be caught up. Identification of people and areas at risk is crucial for efficient confinement. This paper describes a concept which can be applied to healthcare settings. The application skips the time-consuming and imperfect reconstruction of direct and indirect contacts. Indoor mobility of people and devices are instead measured precisely, and the mobility history is used to construct a spatio-temporal ‘landscape of infection’. This landscape allows for the calculation of a modelled ‘contamination landscape’ (CL) adding location-based prolongation of infectivity. In that way, the risk per person can be derived in case of an outbreak. The CL concept is extremely flexible and can be adapted to various pathogen-specific settings. The combination of advanced measurements and specific modelling results in an instant list of possible recipients who need to be examined directly. The modelled, pathogen-specific parameters can be adjusted to get as close as possible to the results of mass screenings.

## 1. Introduction

Despite all efforts to apply standards of hygiene to reduce hospital acquired infections (HAI), infections cannot be avoided altogether, and risk persists, including outbreaks with multiple infections in a hospital [1,2]. This poses a serious health threat to patients, staff and an economic threat to healthcare facilities [3].

Tools for data collection about HAI and various statistics are used to learn about the frequency of HAI outbreaks, their magnitude, possible causes and finally to apply improved hygiene standards and routines to avoid future HAI [2]. Nevertheless, HAI and outbreaks do occur and every outbreak needs an instant strategy for outbreak management [4]. Outbreak management schedules are available from international and national centres for diseases control such as CDC (USA), ECDC (EU) or, for instance, NHS (UK) and RKI (Germany) [5,6,7].

When an outbreak scenario is detected, the pathogen and the infected patient(s) are identified, the phase of the most time-consuming investigation starts: the error-prone collection of information about who has been in contact with infected patients, when, where and how long [8]. The result may be a list of people who are at risk and an area where infection may have taken place. 

In parallel and due to time pressure—since the pathogen already has a head start—possible results are anticipated, and measures are possibly set too small or too large. In the first case, the infection chain is not stopped; the latter threatens the hospital’s capacities and economics [9,10].

This paper proposes a replacement for the investigative tracing of contacts by a four step approach: (i) ongoing measurement of movement profiles; (ii) creation of a spatio-temporal landscape of current presence of infectivity from recorded patients’ movements; (iii) modification of the infection landscape considering room-specific decline of infection potential; and (iv) calculation of all the movements of other people within the modified spatio-temporal landscape of infection, using person-specific probability to acquire an infection within this landscape.

Thus, precise measurements of locations (i) are combined with a pathogen-specific model of infection potential (ii–iv). Measurements are seamless and nearly free of mistakes. They are instantly at hand at any time a threat is detected from the ongoing process of tracking. Pathogen-specific parameters are integrated in the modelling. Every analysis using this concept will provide a list of people at risk which can be compared with the results of mass testing. Thus, the concept’s parameters can be continually improved, gaining relevance and being adapted to local settings.

If a significant period between infection and symptoms has to be considered, the list of people will probably provide information about people who run the risk of already being infected—perhaps without being aware of infection. Possibly, these people are already infectious before any symptoms appear, depending on the pathogen.

The concept is described using a typical hospital background, but with minor modifications it may be adapted to any site where HAI may occur, e.g., homes for the elderly, a sanatorium or a field hospital in cases of emergencies, etc. In any case, one must meticulously comply with the rules of hygiene and this concept is in no way a substitution for thoroughly applied hygiene measures.

## 2. Concept, Materials and Methods

The first step of our concept comprises the precise detection of person-centred in-door location, provided in a dense temporal frequency. Due to energy constraints and the limited technical capacities of earlier devices, such precise detection was difficult when the concept was first presented 15 years ago [11]. Currently, by contrast, the necessary technical background for logistics, healthcare and several other fields of application is provided commercially [12,13,14,15,16,17]. The implementation works silently in the background, hands-free with no interaction necessary by medical staff or patients. Usually, the number of Wi-Fi access points needs to be increased in order to provide access to several access points from each position of a healthcare facility instead of one alone. By using triangulation and comparing results with a previous site-specific calibration (Wi-Fi map), the location of a small device carried by each person can be precisely determined and stored with time, device ID and location in a database [12,18].

The procedure of motion tracking is permanent. The data may be kept for a minimum period, e.g., two days, depending on the timespan between infection and symptoms or the detection of one or more primary index cases (donors). Thus, the spatio-temporal movements of donors are at hand immediately, allowing reconstruction of the infection chain on location without any time-consuming survey.

In case a threat is detected, e.g., at least one person is identified as carrying an infection, it is necessary to determine where this person has been during the previous days, in the event this person has already been in the healthcare facility for a length of time. The person infected may be a patient who is bound to a specific room or bed most of the time, but sometimes patients are mobile, e.g., sent to see a radiation facility in the hospital on their own or allowed to walk around during recovery. However, motion patterns may be much more complex and touch multiple points within a short timespan, allowing contact with many people if the person infected belongs to the medical or technical staff.

The infected person (donor) needs to see a doctor who will perform the diagnosis about the state of illness and determine the possible onset of incubation and infectivity. The estimated onset of infectivity defines the timespan which is extracted for this person from the database of movement patterns, as well as for any other infected person at this state.

The spatio-temporal movements of potential recipients in a hospital are at least as complex as the donor’s movement pattern. Therefore, the possible interactions and contacts are difficult to evaluate, especially if a longer time interval has to be taken into account. Hence, it helps to create an intermediate layer: a so-called spatio-temporal landscape of current presence of infectivity (LPI). The LPI is defined by each location (room) visited by the donor. Once the start and end of the donor’s visit for each room is recorded, we have a list of rooms of interest. Obviously, rooms may have been visited several times. When this list is created, the path itself is no longer of interest: location and timespan are retained; the order of visits and the topology of rooms are dismissed.

Infectivity of people is transferred to and interpreted as infectivity of locations as the result of the presence of an infectious person. This perspective provides advantages when evaluating, e.g., smear infections (see below).

In case the initial donor cannot be identified as one given individual, there may be more than one primary donor: a list of stays of each donor in each room has to be compiled.

Up to this point, all data is a result of measurements and tracing, replacing the formerly necessary personal survey in order to track the movements of patients and staff. Further information on the resulting landscape of infection is now qualified, applying the parameters which are pathogen- and donor-specific.

**Figure 1:**
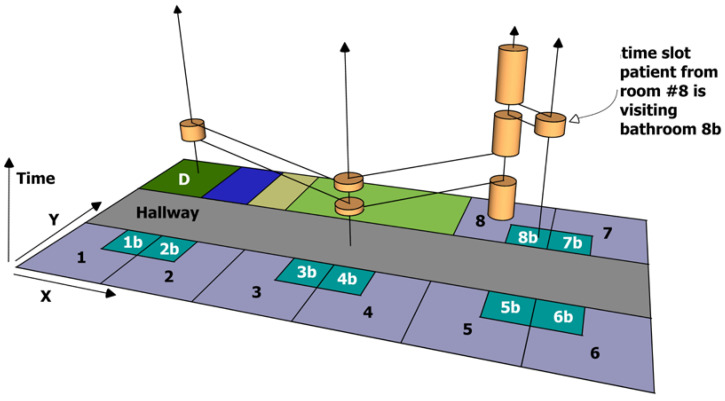
Snapshot from a donor’s moves. Patient from #8 uses the hallway to see his doctor in room D. Afterwards, he returns to his room including a short visit to his bathroom.

The procedure so far is depicted in Figure 1, which displays a very short time span of the patient from room #8, who may be infected and is regarded to be a donor. A potential risk is symbolised by orange pies; their height represents the length of stay in this room. The connecting black lines are representing the path during time. Proceeding with building the landscape of infection, the connecting lines will be dismissed, each room is observed separately with its timeline of risk potentials, without considering near or adjacent rooms. All rooms without a timeline (not visited by donor) are also dismissed.

Any given donor may present an increased likelihood of ‘depositing’ a risk in each of the rooms visited during the period of interest: incontinent people, immunosuppressed patients or mentally disordered people may have greater donor-potential than a young patient with a broken leg or technical staff or educated and risk-aware medical staff, who are used to applying hygiene policies. Additionally, it depends on the pathogen. Therefore, people are classified into donor-qualities importing a specific risk potential for infection into the room during their presence. Tag-IDs are equipped with a donor-quality according to the donor class.

**Figure 2:**
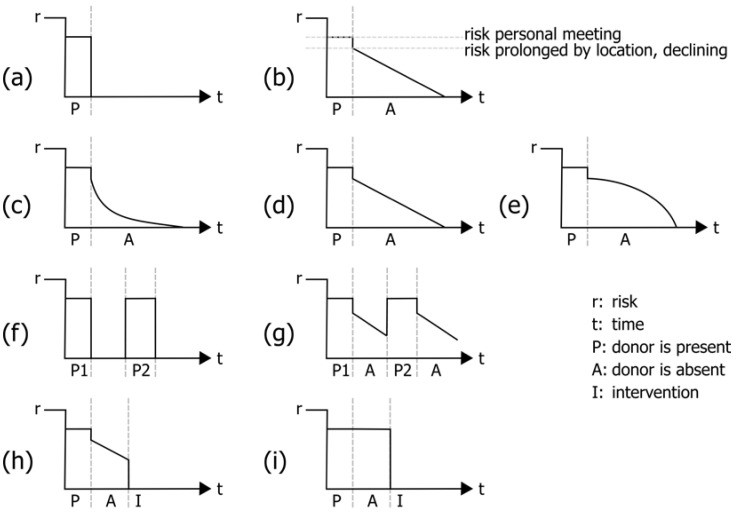
Timelines for infection risk at particular locations and settings. (**a**) infection risk with donor is present, (**b**) ongoing infection risk after donor left a location, (**c**–**e**) different kinds of decay of infection risk in time, (**f**,**g**) risk development of locations revisited by donor, (**h**,**i**) reset of infection risk after intervention at location.

Each room has an individual timeline with individual temporal onset (first visit) and duration (Figure 2a). Rooms may have several visits by donors (Figure 2f), with each room’s timeline commencing individually with the first visit of the first donor up to the present moment.

The rooms are classified as well, since rooms may have different potentials to store and prolong a risk after the donor has left any particular room [19]. In case of the sample ‘norovirus outbreak’, the likelihood of acquiring infection by smear infection has to be taken into account (Figure 2b), while other pathogens may have other implications or possibly will not be extended in a room at all (Figure 2a). Therefore, the risk potential caused by a donor’s visit is room-specific as well as pathogen-specific, e.g., a toilet has a greater potential to extend an infection risk of norovirus than a hallway (Figure 2c–e). The potential will decline over time, but a new visit may reinstate the former risk level (Figure 2g).

To apply this setting to the concept, it is necessary to classify rooms into a short list of types, with respect to the pathogen of interest. Obviously, bathrooms may be important for norovirus infections while influenza will distinguish rooms in other and less extended ways. The basic potential of a room to extend a risk for infection after the donor has left it will usually diminish from the moment when the donor leaves (Figure 2b). Afterwards, different kinds and speeds of decline may be taken into account, reflecting different functions which calculate the remaining risk level forward into the future until the remaining risk level is zero (Figure 2c–e).

After infectivity has been calculated with respect to donor and room potentials to present a risk of infection to a possible visitor (recipient), the resulting ‘contamination landscape’ (CL) is calculated and ready to be evaluated. At this stage, the contamination landscape includes the previously calculated landscape of infectivity and extends this with continuing infectivity without a donor in presence.

The contamination landscape, recorded in a database, consists of rooms showing a risk during the time of interest and risk levels calculated by applying the pathogen-specific function as seen in Figure 2c–e. The people or donors who have imported the risk cannot be observed, so this information is dismissed, as potential risks may have been the result of multiple donors or multiple visits of one donor.

All non-donor tag IDs (people) in the initial database table of movement patterns are now filtered; the temporal aspect needs to be inside the timespan of interest represented in the contamination landscape and rooms visited need to be available in the contamination landscape as well. Recipient’s visits to other rooms are not of interest at this point in time. Along a recipient’s timeline, all visits to rooms in the contamination landscape are summarised, while the room’s risk level at the time of the recipient’s visit is taken into account. The recipient’s ability to acquire an infection is taken into account as a factor, e.g., for immunosuppressed patients it is more likely that the risk potential met will produce an infection than for medical staff, depending on the pathogen [20]. For this reason, recipients are classified, comparable to donors.

This will result into an ordered list of tag IDs with individual level of collected ‘risk levels’ and interpreted as the most probable infected recipients. As the system so far has used tag IDs only, this list needs to be connected to real names, which may be processed manually by a data privacy officer. The people on this list may be infected without actively showing any symptoms and are likely unaware of their status. This situation is comparable to COVID19 tracking apps, e.g., Corona-Warn-App in Germany. Acceptance by users has been described positively in this situation [21] and may be regarded as reasonable for people in a hospital with risk of nosocomial infections, as well.

## 3. Outcome and Options

The results of applying the CL concept provide two major insights within very short time:

(a) A list of people who are likely infected, which provides an idea of the ongoing infection chain and identifies a subset of the people to be taken into account.
(b) A list of rooms having manifested a risk level in the past resulting from the infection chain. This provides an idea of locations to be taken into account, possibly to be closed or disinfected.

These insights are provided on short notice, and they are evidence-based, in contrary to the regular schedule of outbreak management, which includes time-consuming and error-prone surveys about contacts and pure assumptions about areas where the infection may have played a role.

Once testing of identified recipients yields positive knowledge of another infection, the concept needs to be initiated again, now including the new donors as well.

The landscape of infections includes measurements and presence of a donor. The contamination landscapes include additionally modelled extensions of infectivity. There may be pathogens, which depend on direct person-to-person contact, where an extension of infectivity by a location does not apply. In these cases (Figure 2f), the room’s function providing prolonged risk will always return to zero risk. Nevertheless, it is recommended to adhere to the concept’s procedural protocols, as it still may be helpful to model the donor’s ability to spread an infection and recipient’s ability to pick up an infection. 

An advanced modelling may include the length of a donor’s visit as well as a recipient’s length of a visit to a room. In case a potential recipient visits two rooms with comparable infectivity, it is more likely that an infection is acquired on a longer stay than a short drop-in.

A room’s visitor may substantially eliminate a room’s complete infectivity, e.g., if a disinfection takes place or a cleaning personnel is visiting (Figure 2h,i). This type of person may have a negative donor quality, for example, for smear infections of norovirus [22]. Nevertheless, they act as potential recipients and do have a risk of becoming infected themselves. Whether a cleaning personnel is a potential spreader and multiplier (positive donor) or a disinfector (negative donor) may be tested in different scenarios and the concept will show a what-if outcome.

The concept shows in prospect whether the decline of risk is zero (Figure 2i). In this case, the risk once imported by the donor is permanently present, at least until a disinfection takes place. This scenario may be used if extremely infectious situations are of interest, while disinfection is mandatory and anyone visiting this room, intentionally or not, is at high risk.

Tag IDs are meant to be worn by people. Extending this concept, tag IDs may be attached to devices as well, especially if they are directly in contact with a person, e.g., a drug pump or similar. These devices normally must be disinfected before another patient is connected. The devices’ likelihood of ‘acquiring’ an infection is 100%. To make this work, spatial precision must be substantially increased since, for example, a device location in a room with four patients would not reveal which patient is using it. A manual definition of a connection between a patient’s tag ID and device’s tag ID while starting an application may be required [23].

The CL system is meant to be a feedback learning system. The utilised parameters from experiences with this pathogen eventually may not produce a satisfying outcome or the list of potentially infected recipients may not match the result of a mass test. In such a case, the parameters should be modified. 

The selected options described above illustrate the versatility of the concept. However, simple models may be much easier to handle, especially with respect to the learning curve and the adaptation of parameters if the concept’s outcome is compared to the results of mass screening. It is expected that verification and enhancement of the concept by modified parameters is crucial in the initial applications of this method: too many variants integrated just from the beginning may be inefficient. Each parameter and each classification of rooms and people’s needs to be essential for modelling the specific pathogen’s epidemiology, defined and updated with epidemiological evidence and expertise. This is meant to be an expert system and not a sample for machine learning, as is now the case.

## 4. Practical Application

This paper is focused on the theoretical background and the conceptual frame of the CL approach. The following practical considerations comprise the commissioning which allow practical realisation of the concept. Importantly, the steps will need to be discussed with data security officials.

As triangulation of devices on room level will substantially profit from access to more than one access point, existing WLAN installations will probably need improvement. This includes a mapping of WLAN power of accessible access points for every room. WLAN is already present in most of the hospitals; every application supporting the CL concept will benefit from enhancement and investment. Capable logistics manufacturers will be able to provide such services.

Devices need to be purchased and carried by each person: patients, medical staff, other staff, and visitors. Nevertheless, there is no need for additional action by the people who carry devices. Critical medical devices can optionally be tracked, too. Some hospitals may already track devices to locate them in case of emergency. 

The tracking of devices is nothing more than continuously storing the connection of devices on access points with high frequency. A room-based location is then possible using the described WLAN access point mapping.

The resulting database table provides a timestamp, the device’s ID and the room number. Records with time stamps older than three weeks can be deleted automatically. This service is running in the background and does not need any manual intervention or data access.

Only in case of a suspected outbreak will the database table be evaluated. Evaluation will be pathogen-specific, thus a pathogen-specific set of parameters is applied. Parameter sets include:

- the probability of spreading an infection by identified donors (donor-type specific)
- the probability of a room conveying infection potential (room-specific)
- the decline of a potential of infection in time (function, room-specific)
- spatio-temporal contamination landscape (CL)—intermediate result
- the probability of a person acquiring an infection (specific for type of recipients) with respect to location, length of stay and movements in the spatio-temporal CL—final result.

The algorithm to apply the pathogen-specific parameters to the database table holding the information about tracked devices is not yet available. The algorithm itself is not expected to be difficult, as the programme is meant to simply summarise probabilities over time with intermediate declines, finally providing a list of people ordered by their individual risk of infection. 

Due to privacy concerns raised by data security officials, it was not yet possible to achieve an operating mode of the technical setting in a German hospital. As already has been proved for comparable systems elsewhere [24], other national data privacy settings may be expected to allow the implementation of the CL concept and thus efficiently support nosocomial outbreak management.

For the time being, we cannot provide further practical details about an implemented example. Nevertheless, we are convinced that the application of the concept is worth consideration for any healthcare settings. It is relatively inexpensive, easy to realise and safe regarding data privacy. Therefore, we encourage the evaluation of the CL concept as described here, the implementation of automatic movement tracking and the modelling of contamination landscapes.

## 5. Conclusions

The preliminary installation of required hardware has been an obstacle in the past, but nowadays the availability of Wi-Fi may be regarded as ubiquitous in healthcare settings. Precise triangulation allowing for the determination of a single room, or even a part of it in case of a large room, can be realised where several Wi-Fi access points are available providing a sensor network for radio-frequency identification (RFID) tags. Thus, enhanced equipment of Wi-Fi access points may be necessary, together with a spectrum analysis which provides signal strength and connected access points for each location [25]. The technical investment needed, however, is rather limited. 

If a possible threat is detected, the information about movements during an infectious period needs to be at hand or needs to be reconstructed manually. For this reason, the tracking of people necessarily requires data retention, as the event of a future threat is unknown at the time of tracking. To avoid objections concerning data privacy, different aspects which cope with this problem need to be considered:

- The storage of movements has to be secure, human review of data tables must be avoided. The CL concept supports this, as no personal review of data tables is necessary: parameters alone may change, the algorithms can be automated and applied without any manual intervention. The system is able to work like a black box. All recorded data should be deleted automatically when the maximum period of incubation, symptoms and infectivity is reached for the pathogen of interest.
- Tracking is applied to tag IDs only. The connection between the tag ID and a person may be kept undisclosed and available only for a data privacy officer separated from the system. In only two occasions of a concrete threat would this officer intervene: (a) If an infectious risk and a potential donor are identified, the data privacy officer may provide the tag ID of the person (not the person’s name) who is a potential spreader, and (b) After evaluation, the CL system will compile a list of tag IDs which represent people at risk. The data privacy officer may inform these people and invite them to see a physician or to get tested. The evaluating system in-between (a) and (b) does not have access to names or personal identities. Moreover, the database must nonetheless be kept undisclosed.
- Each tag ID is classified and includes the person’s ability to spread or receive an infection, as explained above. This is personal information included in the system. The number of classes needs to be minimised to simplify the modelling in any event. Therefore, as each class will contain numerous people, identification of a single person is not possible.
- The tag ID’s profile of movements may allow identification of a person. This may be problematic, if non-authorised individuals have access to the database. Nevertheless, all people participating need to be informed and consent. Finally, the CL concept presented here provides a quicker and much more efficient means of outbreak management, through protecting people from infection or the need to see a physician before escalation/deterioration. Thus, every participant benefits individually in a setting where risk is high, which may promote participation. The CL system identifies people possibly infected even before they learn of an infection themselves. This opens options for a much easier course of disease, more effective medication and an early recovery.
- The resulting list of tag IDs as well as the resulting list of names (resolved from tag IDs by the data privacy officer) does not allow any reconstruction of the process steps nor does it allow time and place of the possible infection, nor can a connection to a specific donor be determined in retrospect. The resulting list retains a maximum of privacy of the initial donors as well as people wearing a tracking device.

The first and intermediate product of the system named “landscape of current presence of infectivity” is exclusively a direct output from observed movements and does not include assumptions or modelling. This intermediate and transient output may be connected with known movements of any potential recipients, thus providing a simple list of contacts. This preliminary result of tracking is regarded as an immense relief during outbreak management, which otherwise starts with the time-consuming and error-prone task of movement tracking. The tracking and temporary storage of movements explained in this concept takes place completely, instantly and automatically in case of an identified risk.

Additionally, the CL concept explains how to enhance the result by including additional pathogen-specific information. The modification by donor classes, recipient-classes and room classes uses pathogen-specific sets of parameters which support a modelling of risk transport. The resulting contamination landscape is a location-based view of infectivity including advanced temporal aspects.

The term ‘landscape’ suggests a type of mapping. While maps are usually suitable for visualisation, here, the mapping is purely virtual and calculated inside the database, a ‘black box’. On the one hand, extremely complex spatio-temporal aspects are difficult to visualise. On the other hand, such visualisation is not necessary, as we are interested in the result of a spatio-temporal analysis (list of probably infected people), rather than providing an intermediate map. Thus, the privacy of participants is preserved.

Nevertheless, it may be noted that intermediate maps may be of interest, e.g., to optimise sequences of movements by staff or patients, to identify risk spots or to adapt intervals of cleaning and disinfection. These second-use tasks need a specific secure environment, with accompanying supervision by a data privacy officer.

The modelling part of the CL concept is based on pathogen-specific parameters and a small selection of simple functions to project infectivity along a timeline. These parameters can be changed without modifying the backbone of the system, which is a pure database application. Changes should be applied, when the system’s results differ from the results of for example a mass screening. If tag IDs of a mass screening are known, it may be possible to identify rooms which may have been missed or have been attributed with an incorrect parameter. Throughout, the system generates more precision and better output quality in a learning and development process, while this learning aspect—adaption of parameters—requires epidemiological expertise.

## Data Availability

Not applicable.
